# CYP3A and CYP2D6 variability in antimalarial pharmacokinetics and pharmacogenetics across populations: a narrative review

**DOI:** 10.1186/s12936-026-06181-8

**Published:** 2026-10-16

**Authors:** Ursula Benz, Amy Junnila, Günter C. Müller, Patricia Schlagenhauf, Camilla Rothe, Markus Schwarz

**Affiliations:** 1 Institute of Infectious Diseases and Tropical Medicine, LMU University Hospital, LMU Medizin, Ludwig-Maximilians-Universität München, Leopoldstrasse 5, 80802 Munich, Germany; 2 Paleo-DNA Laboratory, Department of Anthropology, Lakehead University, Thunder Bay, Canada; 3 Techniques and Technology of Bamako, Faculty of Medicine and Odonto-Stomatology, University of Sciences, Bamako, Mali; 4 University of Zürich, Institute for Epidemiology Biostatistics and Prevention, WHO Collaborating Centre for Travellers’ Health, Zurich, Switzerland; 5 Institute of Laboratory Medicine, LMU University Hospital, LMU Medizin, Ludwig-Maximilians-Universität München, Munich, Germany

**Keywords:** Antimalarials, Pharmacokinetics, Pharmacogenetics, CYP3A4, CYP3A5, CYP2D6, Quinine, Artemether-lumefantrine, Primaquine, Tafenoquine

## Abstract

Malaria remains a global health burden, requiring optimised antimalarial therapies. This review examines the role of *CYP450 3A* family and *2D6* alleles in antimalarial drug metabolism, highlighting pharmacogenetics and distribution across populations. A narrative literature review was conducted using a structured literature search of PubMed, Web of Science, and Google Scholar to evaluate how *CYP3A* and *CYP2D6* alleles affect the metabolism of quinine, artemether-lumefantrine, primaquine and tafenoquine. The review analysed genetic variations and interactions with other drugs. *CYP3A* alleles influence quinine metabolism, with normally functioning alleles (CYP3A4*1 or CYP3A5*1) associated with higher metabolism rates, while decreased- or non-functional alleles (CYP3A4*1B or CYP3A5*3, *6, *7) are associated with reduced metabolism. The impact of *CYP3A* alleles on artemether-lumefantrine metabolism is less clear but may be relevant in pregnancy, individuals with HIV receiving efavirenz, and individuals with tuberculosis receiving rifampicin. *CYP2D6* alleles associated with poor or intermediate metaboliser phenotypes may contribute to primaquine treatment failure, while interactions with drugs like chloroquine or dihydroartemisinin-piperaquine may elevate primaquine plasma concentrations. Tafenoquine could represent a potential alternative in individuals with reduced CYP2D6 activity, as current human studies have not shown an increased relapse risk, although clinical evidence remains limited. Understanding these genetic differences could help explain variability in antimalarial drug exposure and treatment response across diverse malaria-endemic populations. Genotyping may help explain variability in drug exposure or treatment response in selected clinical settings. Pharmacogenetic insights may therefore support safer and more effective antimalarial therapies worldwide.

**Supplementary Information:**

The online version contains supplementary material available at https://doi.org/10.1186/s12936-026-06181-8.

## Introduction

Research into the pharmacokinetics and pharmacogenetics of antimalarial agents is increasingly recognised as a vital, though still developing, area within malaria research. Key uncertainties remain around mechanisms of action, tolerability, adverse reactions, and drug-drug interactions [1, 2], all of which can differ across populations [3–6]. Treating *Plasmodium falciparum* malaria, often life-threatening if left untreated, carries a heightened risk of drug-related side effects. In the context of travel medicine, these adverse reactions are a major factor in poor adherence to chemoprophylaxis among travellers in endemic regions [7, 8]. Such lapses in adherence contribute to malaria incidence placing travellers at risk of severe disease and even death.

Looking ahead, a central goal for malaria therapy and prevention would be to tailor antimalarial regimens more closely to individual metabolic profiles, adjusting doses accordingly, or to design new agents with improved tolerability [6, 9].

This review aims to provide an overview of the pharmacokinetics, pharmacogenetics, and distribution of genetic variants of relevant cytochrome P450 (CYP) genes across different populations that may influence the efficacy of different antimalarial drugs, offering clinicians insights into the potential implications.

Previous reviews have summarised the pharmacogenetics of antimalarial drugs and CYP pharmacogenes [6, 10]. However, this review provides an updated and clinically focused synthesis by integrating recent evidence on CYP3A4, CYP3A5, and CYP2D6 with population allele distributions, pregnancy, HIV and tuberculosis co-treatment, and recently published studies through June 2026, including dolutegravir co-administration and tafenoquine.

### Brief introduction to relevant CYP enzymes

A wide range of antimalarial agents, whether used preventively or for treatment, are metabolised through the human cytochrome P450 (CYP) enzyme network [3, 4, 6]. Among the CYP enzymes involved in antimalarial drug metabolism, CYP3A4, CYP3A5, CYP2D6, CYP2C8, CYP2C19, CYP2A6, and CYP1A2 are particularly relevant. This review focuses on CYP3A4, CYP3A5, and CYP2D6, for which the strongest pharmacogenetic evidence is currently available [6].

This system consists of membrane-associated haemoproteins with catalytic activity and multiple isoenzymes [11, 12]. CYP enzymes are mixed-function monooxygenases that primarily catalyse the oxidation of lipophilic compounds, thereby increasing their water solubility and promoting their elimination from the body [11]. CYP enzymes catalyse phase I biotransformation reactions, including hydrolysis, dealkylation, deamination, and oxidation, thereby facilitating the metabolism of both endogenous molecules and xenobiotics [6, 11].

Of the 57 known functional human CYP genes, members of CYP families 1, 2 and 3 are particularly important for drug metabolism, with roughly a dozen CYP enzymes accounting for the metabolism of nearly 80% of clinically used drugs [6, 11, 13].

CYP expression and activity are modified by numerous factors, including genetic variation, pharmacokinetic interactions (via induction or inhibition), regulation by cytokines and hormones, disease states, sex, and age [11]. Genetic variation in CYP genes—ranging from single nucleotide variants (SNVs) to insertions, deletions, and copy number changes [6, 14]—often varies across ethnic populations and therefore plays an important role in tropical and travel medicine.

These variations can alter drug-drug-gene interactions, increase susceptibility to adverse reactions, and affect therapeutic outcomes [3–6].

The CYPxyz*aB nomenclature designates cytochrome P450 genes, with x indicating the numerical family, y the lettered subfamily, z the specific gene, *a the allele number, and B a sub-allele designation [6, 15]. The star (*) allele nomenclature system is widely used in pharmacogenetics to describe defined CYP alleles and haplotypes in a standardised and clinically interpretable manner [15]. To support clinical implementation, pharmacogenetic guidelines subsequently translate allele combinations into predicted metaboliser phenotypes based on their functional activity. These phenotypes are commonly classified as poor, intermediate, normal, and ultrarapid metabolisers [16].

### CYP3A4/5

Half of all drugs that are oxidatively metabolised are substrates of the CYP3A family. The predominant hepatic form is CYP3A4, but CYP3A5 also makes a significant contribution to the total human liver CYP3A [17]. CYP3A4 and CYP3A5 overlap in their substrate activity, so that the majority of CYP3A substrates are metabolised by both enzymes [18–20]. This interaction is mostly additive: individuals expressing both *CYP3A4* and *CYP3A5* metabolise substrates much more efficiently than individuals expressing only *CYP3A4* [19, 21].

While the CYP3A5*1 (wild-type) allele is associated with normal CYP3A5 enzyme activity, CYP3A5*3, *6 or *7 are the most common alleles associated with no function [22].

The CYP3A5*1 allele frequency is highest in African populations (~ 56%), followed by Asian populations (~ 30%) and European populations (~ 5%) [23, 24]. In these individuals and in individuals with *CYP3A4* non-functional or decreased-function alleles, *CYP3A5* substantially contributes to metabolism of drugs. This effect is particularly pronounced in drugs with higher affinity to CYP3A5 than to 3A4 [11].

The CYP3A4*1 allele frequency remains consistently high worldwide (~ 95%) [23, 24]. However, the sub-allele CYP3A4*1B, whose function appears to be substrate- and context-dependent, with conflicting evidence between drug classes [25, 26], has been associated with increased enzyme activity in the metabolism of immunosuppressants [27, 28], but appears to exhibit decreased enzyme activity in relation to antimalarials [26]. The CYP3A4*1B allele frequency is highest in African populations (~ 76%), low in European populations (~ 3%), and absent in Asian populations (~ 0%) [29].

### CYP2D6

CYP2D6 is the second most important enzyme in the human CYP system after CYP3A4 and is responsible for the metabolism of approximately 25% of all clinically used drugs [30]. To date, more than 100 CYP2D6 variants have been described, with substantial variation in allele frequencies among different populations [31]. These variants produce substantial interindividual variability in CYP2D6 activity, with consequences for drug exposure and therapeutic response [32].

*CYP2D6* alleles are assigned activity values to standardise genotype-to-phenotype translation. Non-functional alleles receive a value of 0, decreased-function alleles receive activity values of 0.25 or 0.5, and normal-function alleles receive a value of 1.0, with adjustments for gene copy number where applicable. The sum of the activity values determines the activity score (AS), which is translated into the predicted metaboliser phenotype according to the Clinical Pharmacogenetics Implementation Consortium (CPIC) genotype-to-phenotype translation system. Based on the CPIC 2020 recommendations, an AS of 0 defines poor metabolisers (PMs), an AS greater than 0 but less than 1.25 defines intermediate metabolisers (IMs), an AS of 1.25–2.25 defines normal metabolisers (NMs), and an AS greater than 2.25 defines ultrarapid metabolisers (UMs) [16].

Homozygous carriers of normal-function alleles such as CYP2D6*1 and *2 (AS = 2.0) are associated with normal enzyme activity and a normal metaboliser phenotype, whereas alleles such as CYP2D6*4 and *5 (AS = 0) are associated with no-function or decreased-function enzyme activity and a poor metaboliser phenotype [16, 23, 24].

The frequency of CYP2D6 normal metaboliser phenotypes varies substantially across populations, with the highest frequency observed in admixed American populations (~ 73%), followed by European (~ 68%) and East Asian populations (~ 64%), whereas African (~ 38%) and South Asian populations (~ 28%) exhibit considerably lower frequencies [23, 24].

Given these population differences and the availability of established CYP2D6 genotype-to-phenotype translation systems, CYP2D6 phenotype assignment may be relevant to the interpretation of antimalarial treatment response, particularly for drugs whose activation or clearance depends on CYP2D6-mediated metabolism.

### Pharmacokinetic interactions

There are three categories of drugs that influence the CYP enzyme system or are metabolised by them: substrates, inducers and inhibitors [33–35]. CYP substrates are susceptible to drug-drug interactions with inhibitors or inducers of the distinct CYP enzyme. The same CYP substrates may compete in high concentrations for an isoenzyme and can lead to a mutual amplification of effect.

Enzyme inducers can cause loss of efficacy of other drugs or, conversely, can lead to intoxication with a co-administered substrate after discontinuation of the inducer. Enzyme inhibitors can increase the plasma concentration of a co-administered substrate and thus the risk of accumulation and dose-dependent adverse reactions of the co-medication [33].

Unlike active drugs, prodrugs are inactive derivatives of therapeutic substances that gain their biological activity only after being converted within the body by chemical or enzymatic processes. Prodrugs are designed to improve the physicochemical and pharmacokinetic properties of an active drug. However, if CYP enzymes participate in the activation of a prodrug, inhibition may result in less active drug being produced. This can lead to a loss of pharmacodynamic effect. Conversely, induction may result in more active drug being produced. This can lead to an increase in effect [36].

### CYP involvement and the metabolism of antimalarials, used in prophylaxis and therapy

**Fig. 1:**
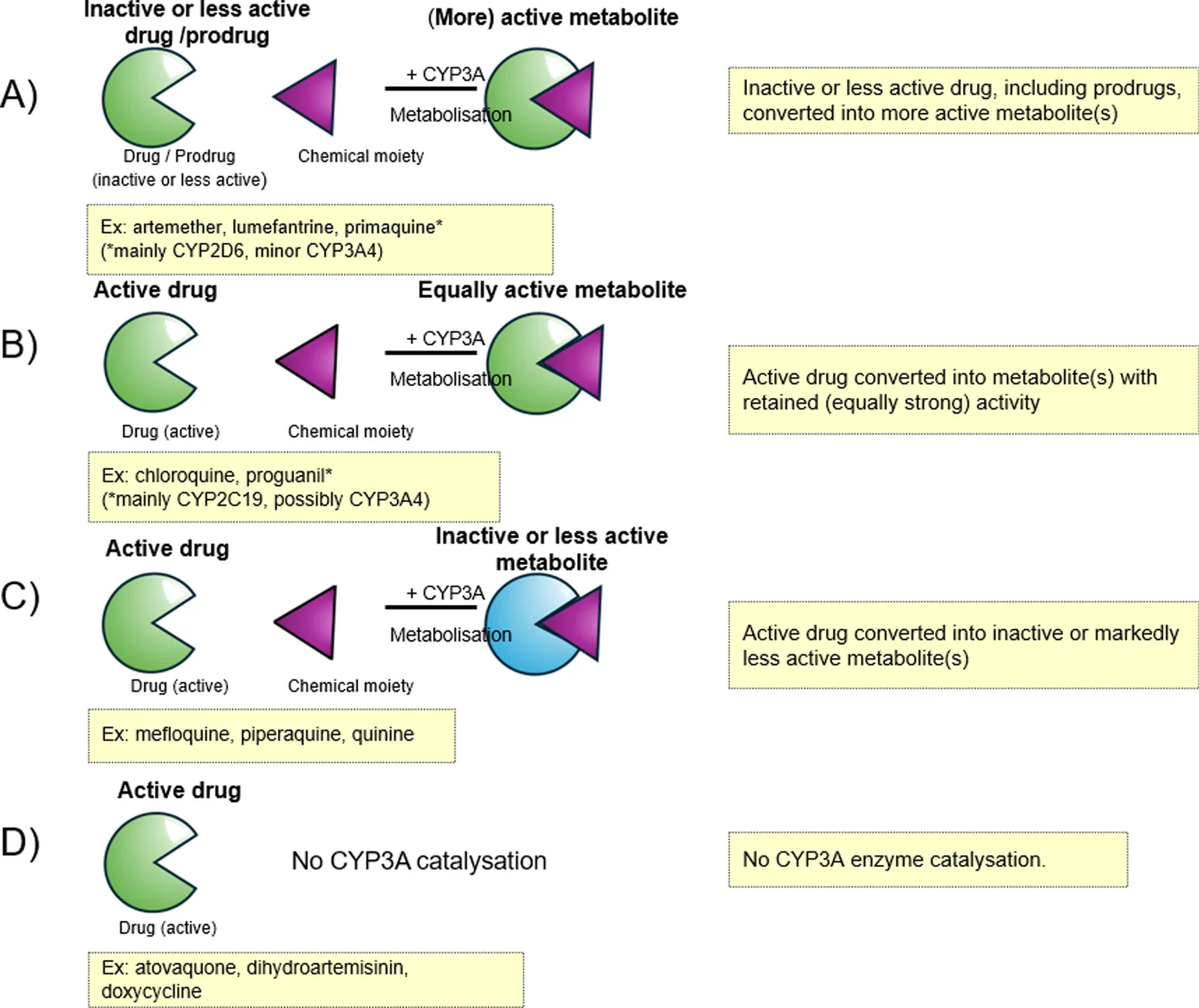
CYP3A-mediated metabolic pathways relevant to antimalarial drugs. Overview of the principal biotransformation routes catalysed by CYP3A enzymes and their impact on antimalarial drug activation, inactivation, and overall pharmacological effect. Four generalised metabolic outcomes are depicted: (**A**) conversion of an inactive or less active drug or prodrug into more active metabolites; (**B**) conversion of an active drug into metabolites with retained (equally strong) activity; (**C**) conversion of an active drug into inactive or markedly less active metabolites; and (**D**) an active drug undergoing no CYP3A-mediated catalysis. Data synthesised from the antimalarial pharmacokinetics and CYP3A metabolism literature cited throughout the manuscript

Antimalarial drugs, including prodrugs, can be converted by CYP enzymes into their metabolites with different activities (Fig. 1).

The metabolism of individual antimalarial drugs involves various CYP enzymes, each contributing to the formation of specific metabolites (Table 1).

**Table 1:** CYP involvement and metabolism of individual antimalarial drugs, developed independently using [6] as a guiding source

Drug (alphabetical order)	CYP Metabolite	CYP involvement	Conversion activity
Artemether	Dihydroartemisinin [37]	Substrate of CYP3A4/5 [38] and to a lesser extent of CYP2B6, 2C9 and 2C19 [6, 39], inducer of CYP2B6, 2C19 and CYP3A activity, inhibitor of CYP1A2 and 2D6 [40, 41]	Artemether is approximately three times less active than dihydroartemisinin, which is the active metabolite [37]
Artesunate	Dihydroartemisinin [42]	Substrate of CYP2A6 [39]	Artesunate is a prodrug and is converted to the active metabolite dihydroartemisinin [39, 42]
Atovaquone	None	None	No evidence that atovaquone is metabolised [43]
Chloroquine	Monodesethyl-chloroquine [39, 44]	Substrate of CYP2C8 and 3A4, moderate inhibitor of CYP2D6 [6, 44]	Monodesethyl-chloroquine has similar antimalarial activity [39, 44]
Dihydroartemisinin	No evidence for clinically relevant CYP450-mediated metabolism [45]	Inhibitor of CYP1A2 [45]	Dihydroartemisinin is the active metabolite of artesunate and artemether [39]
Doxycycline	None	None	Metabolised in the human organism only to a small extent (≤ 10% of a dose) [46]. It is bound to plasma proteins to about 90% [39]
Lumefantrine	Desbutyl-lumefantrine [47]	Substrate of CYP3A4, inhibitor of CYP2D6 [6, 39, 48, 49]	Desbutyl-lumefantrine has an antiparasitic effect in vitro of 5 to 8 times higher than that of lumefantrine [47]
Mefloquine	Carboxy-mefloquine and Hydroxy-mefloquine [50]	Substrate of CYP3A subfamily (mainly 3A4) [6, 39, 50]	Carboxy-mefloquine and Hydroxy-mefloquine are ineffective against *Plasmodium falciparum* [50]
Piperaquine	Carboxylic acid cleavage product and a mono-N-oxidised product [45]	Substrate of CYP3A4 and to a lesser extent CYP2C9 and 2C19, inhibitor of CYP2C19 and 3A4, possible inducer of CYP2E1 [6]	Piperaquine is converted into inactive metabolites [45]
Primaquine	At least six active metabolites [51–53]	Substrate of CYP2D6 and to a lesser extent CYP3A4 and 2C19 [39, 51, 54, 55]	Primaquine is a prodrug, which is converted by CYP2D6 to at least six active metabolites, which are responsible for the anti-hypnozoite activity [51–53]
Proguanil	Cycloguanil [56, 57]	Substrate of CYP2C19 (and possibly via CYP3A4) [6, 39]	Proguanil is effective against malaria, independent of cycloguanil [56, 57]
Quinine	3-Hydroxyquinine [39]	Substrate of CYP3A4/5 and to a lesser extent CYP1A2, 2C9 and 2D6, inhibitor of CYP2D6 [39, 58]	3-Hydroxyquinine contributes around 10% of the antimalarial activity of quinine [39]
Tafenoquine	Metabolism remains uncertain [59–61]	Possibly substrate of CYP2D6 [62]	Tafenoquine appears to act as a prodrug, but a contribution by CYP2D6 on the antimalarial effect is controversially discussed [59, 61, 63]

## Materials and methods

A narrative literature review was conducted using a structured literature search to synthesise the available evidence on antimalarial pharmacogenetics. The final literature search was conducted in June 2026.

Relevant literature was identified through searches of PubMed, Web of Science, and Google Scholar, using the keywords ‘antimalarial drugs’, ‘pharmacogenetics’, ‘pharmacogenomics’, ‘cytochromes’, ‘distribution of cytochrome P450 alleles’, ‘CYP3A4’, ‘CYP3A5’, and ‘CYP2D6’, together with the antimalarial drug names ‘quinine’, ‘artemether-lumefantrine’, ‘primaquine’, and ‘tafenoquine’. Search terms were combined using Boolean operators (AND/OR) and adapted as appropriate for each database. Titles and abstracts were screened for relevance, followed by full-text assessment of potentially eligible articles. In addition, the reference lists of relevant publications were screened to identify further eligible studies.

Pharmacogenetic studies published between January 2005 and the final literature search conducted in June 2026 were eligible for inclusion if they were written in English, peer-reviewed, and explicitly investigated the pharmacogenetics of antimalarial drugs. Earlier foundational publications addressing antimalarial drug metabolism or cytochrome allele distribution were additionally considered where relevant.

To ensure rigour, papers that did not meet the above criteria were excluded. Application of these criteria aimed to ensure the inclusion of relevant peer-reviewed studies. No formal study quality assessment was performed, consistent with the narrative design of this review.

## Results: pharmacokinetic and pharmacogenetic data on the metabolism of antimalarial drugs by CYP3A and CYP2D6 enzymes

The review found that most research on how pharmacokinetics and pharmacogenetics affect CYP3A-metabolised antimalarial drugs focuses on just two medicines: quinine and artemether-lumefantrine (AL). Research on CYP2D6 has focused primarily on primaquine and tafenoquine.

In total, we identified five studies examining CYP3A variation in relation to quinine metabolism, ten studies evaluating CYP3A variation in relation to artemether-lumefantrine, including one population-frequency study, eight studies evaluating CYP2D6 in relation to primaquine, and three studies evaluating CYP2D6 in relation to tafenoquine.

A summary of the expected metabolic consequences of the CYP3A4, CYP3A5, and CYP2D6 variants discussed in this review is provided in a supplementary table (Table S1).

### CYP3A4/5 substrates

#### Quinine metabolism in vivo clinical studies

Pharmacogenetic studies have examined quinine, despite its current relevance being mostly historical due to declining clinical use. Nevertheless, quinine remains a therapeutic option for the treatment of severe *Plasmodium falciparum* malaria. Quinine is also used as a combination therapy with clindamycin during the first trimester of pregnancy or with doxycycline in non-pregnant individuals to treat uncomplicated malaria. Currently, the WHO recommends artesunate for severe malaria and artemether-lumefantrine (AL) for uncomplicated malaria in the first trimester of pregnancy. Quinine is only recommended if artemisinin-based drugs are unavailable [64]. Quinine is an active substrate of CYP3A4 and CYP3A5. Its metabolite, 3-hydroxyquinine, contributes around 10% to the antimalarial activity of the parent compound [39].

Between 2005 and 2010, studies using quinine as a CYP3A probe investigated the interethnic differences in CYP3A activity in different populations by examining the alleles or genotypes of *CYP3A4* and *3A5* and comparing them with CYP3A4/5 catalysed 3-hydroxylation of quinine.

In a Tanzanian population, analysis of *CYP3A4* alleles using quinine as a probe drug in 2005 showed that carriers of the CYP3A4*1B sub-allele had a significantly decreased enzyme activity compared to CYP3A4*1 (wild-type) allele [65].

Two studies from 2006 and 2008, using quinine as a CYP3A probe, demonstrated lower total CYP3A activity in black Tanzanians than in Swedish and Korean populations, indicating interethnic differences in CYP3A activity [66, 67]. Among Tanzanian participants, homozygous carriers of CYP3A5*3, *6 or *7 alleles exhibited significantly higher quinine/3-hydroxyquinine metabolic ratio than heterozygous carriers or homozygous carriers of CYP3A5*1 allele, indicating reduced CYP3A-mediated quinine metabolism [66]. Additionally, Tanzanians who are homozygous for CYP3A5*3, *6, or *7 alleles, and therefore lack extra CYP3A5 metabolic activity, showed lower CYP3A4-catalyzed quinine hydroxylation than Swedes and Koreans. These findings suggest differences in basal CYP3A4 activity between the three populations [66, 67].

A study from Uganda in 2010 also reported significant influence of *CYP3A5* alleles on quinine metabolism variability. There was a significant CYP3A5*1 gene dose effect with higher metabolism. Gender also played a role in this study; women had significantly higher oral quinine bioavailability than men in Uganda [68].

#### Quinine metabolism in vitro

In 2019, an in vitro study on quinine metabolism and *CYP3A4* alleles assessed the enzymatic functions of 31 *CYP3A4* alleles. Among these *CYP3A4* alleles, *23* (CYP3A4*2-*5, *7-*14, *16-*21, *23, *24, and *31-*33) demonstrated significantly decreased enzyme activity, 2 (CYP3A4*15, *29) showed increased activity, 2 (CYP3A4*28, *34) exhibited activity similar to the wild-type CYP3A4*1A, and 3 (CYP3A4*6, *26, *30) showed no enzyme activity. Whether these *CYP3A4* alleles are relevant to the variability of quinine metabolism in vivo can only be confirmed in clinical studies [69].

#### Artemether-lumefantrine (AL) metabolism in vivo clinical studies

Artemether and lumefantrine (AL), in a fixed-dose combination, are used to treat uncomplicated malaria caused by *Plasmodium falciparum*. Since 2022, it has been recommended during all trimesters of pregnancy by the WHO [64]. In 2026, the WHO announced the first artemether-lumefantrine formulation prequalified for neonates and young infants [70]. As CYP3A and CYP2D6 activity undergoes postnatal maturation, pharmacokinetic findings in adults cannot necessarily be extrapolated to neonates and young infants. Further pharmacogenetic studies in paediatric populations are warranted.

Both artemether and lumefantrine are metabolised by CYP3A4 and CYP3A5 enzymes. Artemether is converted to the active metabolite dihydroartemisinin, which is approximately three times more potent than the parent compound [37, 38], whereas lumefantrine, the long-acting component of AL, is converted to desbutyl-lumefantrine, which exhibits an approximately five- to eight-fold greater in vitro antimalarial activity than lumefantrine [47]**.**

##### AL metabolism in non-pregnant patients

A study conducted in 2013 on non-pregnant populations in Tanzania and Cambodia suggested that CYP450 variability did not significantly impact the efficacy of artemisinin-based combination therapy (ACT), including AL. Among 143 evaluable patients receiving AL, no associations were found between lumefantrine pharmacokinetic parameters and CYP3A4*1B or CYP3A5*3 alleles. Likewise, no influence was observed on the pharmacokinetics of mefloquine (n = 63) or piperaquine (n = 60) [71].

Another study, conducted between 2011 and 2013 in 103 non-pregnant uncomplicated malaria patients in Angola, also found no significant association between CYP3A4*1B or CYP3A5*3 alleles and day-28 treatment outcome following AL therapy [72].

One year later, a population-based study in indigenous Tanzanians reported an allele frequency of 78% for CYP3A4*1B and 16% for CYP3A5*3. The authors concluded that further studies are needed to clarify the influence of these variants on the efficacy of artemisinin-based combination therapies and potential drug-drug interactions [73].

In 2024, a study assessed the effect of cytochrome P450 enzymes on the plasma metabolite concentrations of generic AL in non-pregnant Ghanaians. On days 3 and 7, carriers of the CYP3A5*1/*1 genotype showed lower plasma lumefantrine and desbutyl-lumefantrine concentrations compared to other genotypes such as CYP3A5*1/*3, *1/*6, *1/*7, and *3/*6. Statistically significant differences between CYP3A5 genotype groups were observed for desbutyl-lumefantrine on days 3 and 7. Day-7 lumefantrine concentrations remained above the proposed therapeutic threshold, suggesting no clinically relevant reduction in treatment efficacy [74].

##### AL metabolism in pregnancy

Recently, researchers have studied pregnant women who received AL treatment, considering different genotypes of *CYP3A*. A study in 2017 from Tanzania examined the effect of pharmacogenetics on lumefantrine pharmacokinetic parameters and malaria treatment outcome in 92 malaria-positive pregnant women. The study found significant associations between *CYP3A5* genotypes and day-7 plasma lumefantrine concentrations. They were significantly higher and were therefore associated with lower metabolism in homozygous and heterozygous carriers of CYP3A5*3, *6 or *7 alleles than in homozygous carriers of CYP3A5*1 allele (*1/*1 genotype) [75]. Furthermore, the study showed that the majority of pregnant women with CYP3A4*1B/*1B genotype had a significantly better adequate clinical and parasitological response (ACPR) than those with CYP3A4*1/*1 genotype after observation and examination on day 28 [75]. The authors emphasise the importance of these findings, as genotyping could potentially be used in the future to predict the need for antimalarial drug dosage adjustments in pregnant women with the CYP3A4*1/*1 or CYP3A5*1/*1 genotypes [75].

An expanded follow-up study in 2019 from Tanzania involved 205 pregnant and 72 non-pregnant women. The study investigated the impact of pregnancy and genotyping on day-7 lumefantrine concentration and malaria treatment outcomes. The results revealed significantly lower day-7 lumefantrine concentrations in women with pregnancy, low body weight, and CYP3A5*1/*1 genotype. The authors stated that low day-7 lumefantrine plasma concentration significantly predicts treatment failure in pregnant women [76].

##### AL metabolism in HIV patients

HIV-malaria co-infected patients co-treated with AL and efavirenz-based combination antiretroviral therapy (cART) show significantly reduced artemether and lumefantrine plasma concentrations, as efavirenz is a CYP2B6 and CYP3A inducer [4, 77]. In 2016, researchers in Tanzania examined how *CYP2B6*, *CYP3A4*, and *CYP3A5* alleles affected lumefantrine plasma concentrations on day 7 among HIV-positive patients. Of the 269 patients enrolled by Maganda et al., the primary analysis focused on 60 individuals receiving efavirenz-based cART and 70 cART-naive controls. The CYP2B6*6/*6 genotype and the use of efavirenz were significantly correlated with low lumefantrine plasma levels and an elevated incidence of recurrent parasitaemia. However, CYP3A4*1B and CYP3A5*3 alleles showed no significant influence on the day-7 lumefantrine concentrations [78]. These findings suggest that, in this study population, CYP3A induction by efavirenz had a greater influence on lumefantrine exposure than CYP3A4*1B and CYP3A5*3 alleles.

In contrast, a 2020 study from Nigeria investigated the effect of CYP3A5*3 allele on lumefantrine plasma concentrations among 69 HIV-malaria co-infected women receiving ongoing efavirenz-based cART. The findings indicated that the CYP3A5*3 allele is associated with a high maximum plasma concentration of lumefantrine. Plasma levels of lumefantrine on day 7 were not considered. The authors suggest further studies to explore the relationship between CYP3A5*3 allele, the pharmacokinetics of lumefantrine, and the electrophysiological effect, as higher plasma exposure to lumefantrine might contribute to prolongation of the QTc interval as an adverse reaction [79].

Since 2019, the integrase strand transfer inhibitor dolutegravir, which is metabolised primarily by UGT1A1 and to a lesser extent by CYP3A4 [80], has replaced efavirenz-based cART as the preferred first-line HIV treatment [81], and is recommended in pregnancy [82]. However, the WHO still recommends efavirenz as an alternative first-line regimen for adults and adolescents [81].

A 2025 Ghanaian pharmacogenetic study reported reduced plasma exposure of both dolutegravir and artemether-lumefantrine during co-administration in HIV-malaria coinfection, with lower day-7 plasma concentrations of lumefantrine (426.0 vs. 741.5 ng/mL; 57–66% reduction across sampling time points) and dolutegravir (134.85 vs. 162.5 ng/mL). Reduced drug concentrations were observed in carriers of CYP2B6*18, CYP3A4*1B, and CYP3A5*3, *6, and *7 variant alleles. The reduction was more pronounced for artemether-lumefantrine, whereas dolutegravir remained largely above the protein-adjusted IC90, indicating preserved antiviral exposure. No clinically significant adverse reactions were observed, although a potential risk of subtherapeutic exposure in coinfection and variant carriers was noted [83].

##### AL metabolism in patients with tuberculosis

Tuberculosis-malaria co-infected patients undergoing co-treatment with AL and rifampicin-based therapy, as part of the tuberculosis treatment [6], show a significant reduction in artemether and lumefantrine plasma concentration. This decrease is due to rifampicin acting as an inducer of CYP3A [84, 85]. To date, there is a lack of direct studies on concomitant treatment with rifampicin and lumefantrine and the effects of different *CYP3A* alleles or genotypes.

#### AL metabolism in vitro

In 2023, an in vitro study on artemether-lumefantrine metabolism and *CYP3A4* alleles assessed enzymatic activity. Compared to CYP3A4*1 (wild-type) allele, CYP3A4*4, *5, *9, *16, *18, *23, *24, *28, and *31-*34 alleles demonstrated significantly decreased enzyme activity in metabolising artemether. Conversely, CYP3A4*11, *15, *18, *19, *23, *28, *29, and *31-*34 alleles showed significantly increased enzyme activity in metabolising lumefantrine, while CYP3A4*5, *14, *17, and *24 exhibited decreased enzyme activity compared to CYP3A4*1 (wild-type) allele. The CYP3A4*18, *23, *28, and *31-*34 alleles exhibited completely opposite metabolic functions [86].

### CYP2D6 substrates and their effect on primaquine and tafenoquine

Primaquine is used as a radical cure to prevent relapses in case of *Plasmodium vivax* and *Plasmodium ovale* malaria for individuals without glucose−6-phosphate dehydrogenase (G6PD)-deficiency, which may cause haemolysis [87, 88]. In the United States, it is also used for primary prophylaxis in regions where *Plasmodium vivax* malaria predominates [64, 89]. It is an 8-aminoquinoline prodrug, which is converted by the CYP2D6 enzyme to at least six active metabolites responsible for the anti-hypnozoite activity [51–53]. Recent studies suggest that reduced CYP2D6 activity, particularly in poor and intermediate metabolisers, may contribute to primaquine treatment failure [52].

Both primaquine treatment failure reports and a malaria mouse model support an association between reduced CYP2D6 activity (activity score (AS) ≤ 1.0) and primaquine treatment failure [55, 90–92]. In addition, reduced CYP2D6 activity was associated with a significantly higher risk of multiple *Plasmodium vivax* recurrences (RR 1.75, 95% CI 1.2–2.6; p = 0.0035) [92].

A 2021 Brazilian case series reported three patients with *Plasmodium vivax* malaria suffering repeated relapses despite treatment with chloroquine and primaquine. After the primaquine dosage was increased, two patients did not experience any additional relapses. One patient experienced two further relapses and was given an additional 300 mg of chloroquine weekly to help prevent further episodes. Reduced CYP2D6 activity (AS ≤ 1.0) was observed in all three patients, including one poor metaboliser, one intermediate metaboliser, and one normal-slow metaboliser [93].

A 2025 study from northeastern Myanmar found that over 75% of patients with *Plasmodium vivax* malaria carried *CYP2D6* alleles associated with reduced metabolic phenotype (AS ≤ 1.25), corresponding to normal-slow, intermediate, or poor metaboliser status. This was significantly associated with a higher risk of recurrence (OR 6.53; 95% CI 2.0–21; p < 0.001) after standard primaquine treatment. The authors suggested that a higher dose adjustment may be required in individuals with reduced enzymatic activity [94].

A Brazilian Amazon study also from 2025 found that the no-function CYP2D6*4 allele (AS = 0) was more frequent among recurrence cases, while mutated *MAOA* genotypes were associated with shorter time to *Plasmodium vivax* recurrence following primaquine treatment. CYP2D6 normal metaboliser phenotypes were more frequent in non-recurrent cases [95].

In contrast, a Colombian study published in the same year on *Plasmodium vivax* malaria treatment with chloroquine-primaquine found that carriers of *CYP2D6* alleles associated with reduced metabolic phenotype (AS ≤ 1.25) showed higher day-2 primaquine plasma concentrations, although this difference was not statistically significant, and relapse risk was not significantly increased compared with normal or ultrarapid metabolisers. Notably, 18% of participants had a reduced CYP2D6 metabolic phenotype [96].

Not only genetic variations, but also interactions between drugs could result in clinically relevant pharmacokinetic alterations. Co-administered drugs such as chloroquine, a CYP2D6 inhibitor, and dihydroartemisinin-piperaquine can significantly increase primaquine plasma concentrations [97–99].

Tafenoquine was authorised by the Food and Drug Administration (FDA) in the United States in 2018 and has also been approved in multiple other countries since then. It has no approval from the European Medicines Agency (EMA), nor is there any pending application for EMA approval. Tafenoquine is used for prophylaxis against all *Plasmodium* spp. in malaria-endemic regions and for radical cure of *Plasmodium vivax* malaria. Quantitative G6PD testing is required before tafenoquine administration [100, 101]. It is contraindicated for individuals with G6PD deficiency or unknown G6PD status [100]. In addition, tafenoquine is currently subject to age restrictions, being approved for radical cure in patients aged ≥ 16 years and for malaria prophylaxis in adults aged ≥ 18 years. It is contraindicated during pregnancy and during breastfeeding when the infant's G6PD status is deficient or unknown [101]. Despite the fact that the mechanism of action of the drug remains unknown, research findings consistently indicate that a metabolite is responsible for the drug’s efficacy [59]. However, recent human studies suggest that reduced CYP2D6 activity is not associated with an increased risk of relapse following tafenoquine treatment [61, 63, 102].

## Discussion

This review focuses on the current state of knowledge in pharmacogenetics regarding the impact of drug metabolism via CYP enzymes in antimalarial therapy, and provides clinicians with an overview of its potential clinical implications. The included studies were conducted across diverse geographic regions, including Africa, Asia, Europe, and South America, highlighting population-specific differences in CYP allele distribution and antimalarial pharmacogenetics.

The investigation revealed that pharmacogenetic studies on antimalarials and *CYP3A* alleles in different populations have primarily focused on quinine and artemether-lumefantrine.

*CYP3A* alleles influence the metabolism of quinine. Several studies showed that CYP3A4/5-catalysed 3-hydroxylation of quinine exhibits interethnic differences in CYP3A enzyme activity between different populations [66, 67].

Homozygous carriers of CYP3A5*1 (wild-type) normal function allele exhibit a high metabolism rate of quinine to its less active metabolite, 3-hydroxyquinine [66–68]. In contrast, reduced-expression *CYP3A5* alleles (*3, *6 or *7) were associated with progressively lower quinine metabolism, consistent with a *CYP3A5* gene-dose effect [66–68]. The highest frequencies of CYP3A5*1 have been reported in sub-Saharan African populations, whereas substantially lower frequencies are observed in European populations and intermediate frequencies in East Asian populations. Interethnic variation in CYP3A5*1 allele frequency is considerable, with the highest frequency reported in African populations (~ 56%), followed by Asian (~ 30%) and European populations (~ 5%) [23, 24].

A comparable trend was found for the CYP3A4*1 allele and the CYP3A4*1B sub-allele. Carriers of the CYP3A4*1B sub-allele exhibited decreased enzyme activity and lower quinine metabolism than carriers of the CYP3A4*1 allele [65]. However, the functional consequences of CYP3A4*1B appear to be substrate- and context-dependent, with conflicting evidence reported across different drug classes [25–28]. CYP3A4*1B allele frequencies differ markedly across populations, with the highest frequency in African populations (~ 76%), compared with European populations (~ 3%), and the allele is virtually absent in Asian populations (~ 0%) [29].

The lower CYP3A4-catalysed quinine metabolism observed in Tanzanians homozygous for CYP3A5*3, *6, or *7 alleles compared with Swedish and Korean populations suggests that this effect is unlikely to be explained solely by the CYP3A4*1B sub-allele [66, 67]. Additional genetic factors may contribute to the observed differences in CYP3A activity. Recently identified *CYP3A4* alleles, many of which are associated with reduced enzyme activity [69], may partly account for this variability.

In clinical terms, a CYP3A5 normal metaboliser phenotype may contribute to interindividual variability in quinine exposure. However, current evidence is insufficient to recommend genotype-guided quinine dose adjustment, and prospective clinical studies are required before such an approach can be considered. Given the additive role of CYP3A4 and CYP3A5 in quinine metabolism, reduced activity of one enzyme may be partially compensated by the other. However, if patients do not respond to treatment with quinine, genotyping may help identify underlying causes of variability in quinine exposure.

Evidence for artemether-lumefantrine (AL) is less consistent than for quinine. Data remain limited, and most studies include small sample sizes. The metabolism of AL, a fixed-dose combination, is complex, as both artemether and lumefantrine are converted via CYP enzymes into more active metabolites, whereas quinine is converted into the less active metabolite 3-hydroxyquinine [38, 39, 47]. This complicates interpretation of pharmacogenetic effects and treatment outcomes. Most studies on AL focused more on lumefantrine, the long-acting component of AL.

In addition to pharmacogenetic variation, lumefantrine exposure is influenced by several important non-genetic factors, including food intake, body weight, treatment adherence, and sampling time. These factors may substantially contribute to pharmacokinetic variability and should therefore be considered alongside CYP3A genetic variation when interpreting lumefantrine concentrations and treatment outcomes [103].

Overall, the data suggest that CYP3A pharmacogenetic variation has limited impact on AL efficacy [10] in non-pregnant patients in different populations [71, 72]. A study on non-pregnant patients in Ghana reported lower plasma concentrations of generic lumefantrine and desbutyl-lumefantrine on days 3 and 7 after intake in CYP3A5*1/*1 carriers compared to other genotypes, including CYP3A5*1/*3, *1/*6, *1/*7, and *3/*6. Statistically significant differences between genotype groups were reported for desbutyl-lumefantrine on days 3 and 7; however, lumefantrine concentrations remained above the therapeutic threshold (≥ 200 ng/mL) in all groups [74].

Generally, lower plasma exposure of lumefantrine has been observed in pregnancy [104–106] and young children [107], as well as during co-treatment with efavirenz-based antiretroviral therapy of HIV [4, 77, 78] or rifampicin-based tuberculosis treatment, both of which induce CYP3A4/5 [84, 85].

Associations between different *CYP3A* genotypes, day-7 plasma concentrations of lumefantrine and treatment outcomes at day 28 have been reported in pregnancy. Lower lumefantrine levels on day 7 were associated with increased treatment failure risk in pregnant women [75, 76].

Therapeutic drug monitoring (TDM) may complement pharmacogenetic testing by reflecting actual drug exposure and the combined effects of genetic and non-genetic factors [6, 11, 108]. However, TDM results may be influenced by factors such as patient adherence, sampling time, and pharmacokinetic variability [108].

Generally, day-7 lumefantrine concentrations have been proposed as a practical predictor of treatment response, as they correlate more strongly with outcomes than area under the curve estimate [109]. However, other studies have reported no significant association between day-7 lumefantrine concentrations and treatment outcomes in children [110–112] or non-pregnant adults [112, 113]. In pregnant women, day-7 lumefantrine concentrations were found to be up to approximately 34% lower compared to non-pregnant women [104–106].

Although CYP3A4/5 activity may increase during pregnancy [114, 115], malaria infection itself induces a dysfunction in hepatic mixed-function oxidase activity, leading to elevated levels of acute-phase proteins and inflammatory cytokines, which reduce CYP-mediated clearance of antimalarials and other drugs [116, 117]. In this context, higher therapeutic thresholds (≥ 600 ng/mL on day 7) have been proposed for pregnant patients compared with non-pregnant adults (175–280 ng/mL) [118]. However, these findings should be interpreted with caution. Previous authors have highlighted methodological limitations in several pregnancy pharmacokinetic studies, including challenges related to study design, sampling strategies, and the interpretation of pregnancy-associated pharmacokinetic changes [119].

In HIV-malaria co-infected women undergoing co-treatment with AL and efavirenz-based combination antiretroviral therapy (cART), the CYP3A5*3 allele has been associated with elevated lumefantrine maximum plasma concentrations [79]. Concomitant administration of dolutegravir and artemether-lumefantrine in HIV-malaria coinfection was associated with reduced plasma drug exposure, as evidenced by lower day-7 lumefantrine concentrations and decreased dolutegravir levels. Lower drug concentrations were observed in carriers of CYP2B6*18, CYP3A4*1B, and CYP3A5*3, *6, and *7 variant alleles [83].

Taken together, these findings suggest that CYP3A induction by efavirenz may have a greater impact on lumefantrine exposure than CYP3A genetic variation alone. Drug-drug interactions may therefore outweigh genetic effects in HIV-infected patients receiving efavirenz-based cART. However, further prospective studies are needed to clarify the relative contribution of genetic and non-genetic factors. The concomitant reduction in dolutegravir and artemether-lumefantrine exposure may reflect a complex interaction between CYP3A induction, co-medication, and genetic variation. However, the underlying mechanisms remain unclear.

Regarding tuberculosis-malaria coinfection, no data are currently available on the influence of CYP3A pharmacogenetic variants during co-treatment with AL and rifampicin-based therapy.

However, both efavirenz and rifampicin are potent CYP3A inducers, leading to reduced plasma exposure to the CYP3A substrates artemether and lumefantrine. Artemether is bioactivated to the active metabolite dihydroartemisinin, whereas lumefantrine is metabolised to desbutyl-lumefantrine, which exhibits greater in vitro antimalarial activity than the parent compound. In a study on efavirenz-based cART, lumefantrine levels decreased by 47%, while rifampicin therapy resulted in a 59% reduction due to CYP3A4 induction. The probability of day-7 lumefantrine concentrations below 200 ng/mL, a threshold associated with an increased risk of treatment failure, was estimated at 49% with efavirenz therapy and 80% with rifampicin therapy [120]. In contrast, the CYP3A4 substrate dolutegravir [80] co-administration with artemether-lumefantrine does not generally require dose adjustment in standard regimens [121, 122]. Nevertheless, the observed reductions in AL and dolutegravir exposure during co-administration may warrant closer pharmacokinetic evaluation in vulnerable populations, including pregnant women and patients with high baseline viral loads.

These observations suggest that pharmacogenetic effects on lumefantrine may only become clinically relevant under conditions of additional metabolic stress, such as pregnancy or concomitant CYP3A induction. Further studies across diverse populations are required, particularly in pregnant women, HIV-infected patients on efavirenz-based cART, and tuberculosis patients receiving rifampicin. Further pharmacogenetic evaluation of dolutegravir co-therapy is also warranted given its increasing global use.

In clinical terms, for selected high-risk patients, such as pregnant women or patients receiving CYP3A-inducing co-medication, therapeutic drug monitoring of lumefantrine may be considered in specialised clinical or research settings to support the interpretation of treatment failure or unexpected drug exposure. However, routine therapeutic drug monitoring cannot currently be recommended. Genotyping may be useful in selected cases of unexplained treatment failure. In African populations, where CYP3A5*1 allele frequency is higher than in European populations, CYP3A5-mediated lumefantrine metabolism may contribute to variability in drug exposure [23, 24].

For malaria prevention in pregnant women living in areas of moderate-to-high *Plasmodium falciparum* transmission, the WHO recommends intermittent preventive treatment with sulfadoxine-pyrimethamine (IPTp-SP), starting in the second trimester [64]. However, increasing sulfadoxine-pyrimethamine resistance has prompted the evaluation of dihydroartemisinin-piperaquine as a potential alternative IPTp regimen in clinical studies [123]. Although these studies have shown promising antimalarial efficacy, current evidence remains insufficient to recommend routine replacement of IPTp-SP [64, 123].

The drugs primaquine and tafenoquine, primarily used for radical cure and relapse prevention in *Plasmodium vivax* malaria, require prior exclusion of glucose−6-phosphate dehydrogenase (G6PD) deficiency. Primaquine is a prodrug metabolised by the CYP2D6 enzyme into several active metabolites [51–53]. Tafenoquine metabolism remains uncertain, however, it also appears to be a prodrug converted by CYP2D6 to its active metabolite [59], at least in mouse models [62, 124]. Tafenoquine has a prolonged half-life of 15 days [60], compared with a few hours for primaquine [39]. Treatment with tafenoquine only requires a single-dose regimen, whereas primaquine necessitates a seven-day course or weekly doses over 8 weeks [125].

Reduced CYP2D6 activity has been associated with primaquine treatment failure and *Plasmodium vivax* recurrence in several clinical studies, particularly in poor and intermediate metaboliser phenotypes [52, 55, 90–95]. In contrast, recent research has indicated that these CYP2D6 phenotypes do not appear to be associated with treatment failure in tafenoquine [61, 63, 102].

A 2024 Brazilian study demonstrated that tafenoquine was more effective than primaquine in preventing malaria recurrence by day 90 in G6PD-normal patients aged ≥ 16 years. By day 180, both treatments showed similar effectiveness. These findings suggest that the *Plasmodium vivax* treatment plan could be used in Brazil and other South American countries [125]. Additionally, modelling studies indicate that tafenoquine administration following G6PD testing is cost-effective in Brazil [126].

If confirmed in further studies, tafenoquine could serve as an alternative to primaquine in individuals with reduced CYP2D6 activity, although clinical evidence remains limited. Its single-dose regimen enhances convenience, and if cost-effectiveness is established, it may become an attractive alternative. However, tafenoquine has not yet received approval in several countries, including those in Europe.

### Study strengths and limitations

This narrative review has several limitations. Although the literature search was structured, the review was not conducted as a systematic review and is therefore subject to selection bias. In addition, no formal study quality or risk-of-bias assessment was performed. The available pharmacogenetic studies are heterogeneous with regard to study design, populations, pharmacokinetic sampling strategies, genotype definitions, and outcome measures, and evidence remains limited for several antimalarial agents. Although key quantitative findings were incorporated wherever available, the heterogeneity of study designs, pharmacokinetic endpoints, and reported outcome measures precluded formal quantitative comparison or meta-analysis. Furthermore, several pharmacogenetic associations are based on relatively small studies in specific populations, which may limit their generalisability. Consequently, some findings should be interpreted with caution and require confirmation in larger prospective studies.

## Conclusions

Pharmacogenetic variation contributes to variability in antimalarial drug metabolism, particularly through CYP3A and CYP2D6 pathways. Quinine and artemether-lumefantrine are the most extensively studied antimalarials in relation to CYP3A4 and CYP3A5 variants. Evidence demonstrates substantial interethnic differences in quinine metabolism associated with CYP3A alleles, which may contribute to interindividual variability in quinine exposure. However, current evidence is insufficient to support routine genotype-guided quinine dose optimisation. Although CYP3A alleles appear to have limited impact on artemether-lumefantrine efficacy in non-pregnant populations studied to date, CYP3A activity may be altered during pregnancy or by co-medication, particularly HIV treatment with efavirenz and tuberculosis treatment with rifampicin. These modifiers highlight the need for careful therapeutic management in these high-risk groups and underscore the potential clinical relevance of CYP3A-mediated variability in real-world malaria treatment settings.

Reduced CYP2D6 activity, particularly poor and intermediate metaboliser phenotypes, has been associated with reduced primaquine efficacy and an increased risk of *Plasmodium vivax* recurrence in several clinical studies. Recent research indicates that these phenotypes do not appear to affect the risk of tafenoquine treatment failure. These findings support a potential contribution of *CYP2D6* genetic variation to primaquine efficacy across different populations and highlight the importance of understanding CYP2D6 phenotype distributions. Furthermore, tafenoquine may represent a potential alternative in individuals with reduced CYP2D6 activity, although evidence remains limited.

Overall, the evidence highlights the importance of considering the pharmacogenomic context—including allele frequencies, co-morbidities, co-medications, and physiological states—when interpreting antimalarial pharmacokinetics and treatment variability. Clinicians should be particularly aware of pregnancy, HIV or tuberculosis co-treatment, recurrent malaria despite standard therapy, and potential drug-drug interactions, as these factors may substantially alter lumefantrine exposure and antimalarial treatment outcomes. In selected cases, *CYP2D6* genotyping may help explain recurrent *Plasmodium vivax* malaria following primaquine treatment, while therapeutic drug monitoring may support the evaluation of unexpected lumefantrine exposure or treatment failure in selected high-risk patients. Although routine pharmacogenetic testing is not yet standard, integrating these insights into clinical decision-making may help clarify unexplained treatment failures and support more individualised malaria management. Complementary strategies, including therapeutic drug monitoring in selected high-risk patients or specialised clinical settings, targeted genotyping, and refined clinical algorithms, should be further evaluated for their potential to improve treatment safety and effectiveness. These approaches are particularly relevant across diverse malaria-endemic populations or travel medicine settings, where treatment variability and vulnerability are highest.

By integrating these findings, this review clarifies the role of CYP3A and CYP2D6 pharmacogenetics in antimalarial therapy and provides a framework for further research into personalised antimalarial treatment strategies and pharmacogenetically informed malaria control approaches.

## Supplementary Information

Additional file 1

## Data Availability

No datasets were generated or analysed during the current study.

## Abbreviations

ACT: Artemisinin-based combination therapy
AL: Artemether-lumefantrine
AS: Activity score
cART: Combination antiretroviral therapy
CYP: Human cytochrome P450
EMA: European Medicines Agency
G6PD: Glucose−6-phosphate dehydrogenase
IPTp-SP: Intermittent preventive treatment of malaria in pregnancy with sulfadoxine-pyrimethamine
TDM: Therapeutic drug monitoring
WHO: World Health Organisation
