# Supplementary material for: CYP3A and CYP2D6 variability in antimalarial pharmacokinetics and pharmacogenetics across populations: a narrative review
Source: Malar J. 2026 Oct 16;25:368. doi: 10.1186/s12936-026-06181-8 (PMC13675977; doi:10.1186/s12936-026-06181-8)
Supplement: Supplementary file 1 [file 12936_2026_6181_MOESM1_ESM.docx]

**CYP3A and CYP2D6 Variability in Antimalarial Pharmacokinetics and Pharmacogenetics Across Populations: A Narrative Review**

**Supplementary data**

**Table S1.** **Overview of CYP3A4, CYP3A5 and CYP2D6 variants and their expected impact on antimalarial drug metabolism.**

| Expected or reported direction of metabolic activity associated with CYP alleles and antimalarials | *CYP3A4*1*  Normal-function allele | *CYP3A4*1B*  Decreased-function allele regarding antimalarials | *CYP3A5*1*  Normal-function  allele | *CYP3A5*3, *6, *7*  No-function alleles | *CYP2D6*  NM^2^ | *CYP2D6*  IM^1^, PM^3^ |
| --- | --- | --- | --- | --- | --- | --- |
| Quinine  converted into less active metabolite | higher | reduced | higher | reduced | - | - |
| Lumefantrine  converted into more active metabolite | higher | reduced | higher | reduced |  |  |
| Primaquine  (prodrug) converted into active metabolites | - | - | - | - | higher | reduced, low |

1)IM= intermediate metaboliser phenotype, 2)NM= normal metaboliser phenotype, 3)PM= poor metaboliser phenotype

**Table S2. Characteristics and principal findings of studies investigating CYP3A4, CYP3A5 and CYP2D6 in relation to quinine, artemether-lumefantrine, primaquine and tafenoquine.**

| Study (reference) | Country / population | Antimalarial drug | CYP genotype / phenotype | Study design | Primary outcome | Principal finding / direction of effect |
| --- | --- | --- | --- | --- | --- | --- |
| **3.1.1 Quinine metabolism in vivo clinical studies** | | | | | | |
| Rodríguez-Antona et al. (2005) (65) | Tanzania; 143 healthy volunteers | Quinine (single probe dose) | *CYP3A4*1B* versus *CYP3A4*1* | Pharmacogenetic phenotype-genotype association study | CYP3A activity assessed using quinine as a probe | *CYP3A4*1B* carriers had significantly lower CYP3A activity than *CYP3A4*1* carriers; five other investigated *CYP3A4* variants showed no significant association. |
| Mirghani et al. (2006) (66) | Tanzania (n=144) and Sweden (n=136); healthy volunteers | Quinine hydrochloride, single 250-mg oral dose | *CYP3A5* high-expression versus low-expression alleles *(*1, *3, *6, *7* as population-relevant) | Comparative pharmacogenetic phenotyping study | Plasma quinine/3-hydroxyquinine metabolic ratio 16 h after dosing | Within Tanzanians, high-expression *CYP3A5* alleles were associated with a lower quinine/3-hydroxyquinine ratio (greater hydroxylation). Overall, Tanzanians had a higher ratio than Swedes, indicating lower total CYP3A activity despite more frequent CYP3A5 expression. |
| Diczfalusy et al. (2008) (67) | Tanzania (n=138), Sweden (n=161) and Korea (n=149); healthy volunteers | Quinine probe | *CYP3A5* genotype; sex | Comparative clinical phenotyping study | 4β-Hydroxycholesterol, quinine 3-hydroxylation and sex | 4β-Hydroxycholesterol was evaluated as an endogenous CYP3A marker. CYP3A activity differed across populations and by sex; genotype did not fully explain the interpopulation differences. |
| Mukonzo et al. (2010) (68) | Uganda; 140 healthy volunteers | Quinine, single 600-mg oral dose | *CYP3A5*1* and *ABCB1* variants; sex | Clinical pharmacokinetic/pharmacogenetic study | Quinine disposition, metabolism and oral bioavailability | *CYP3A5*1* showed a gene-dose association with greater quinine metabolism. Sex also influenced disposition; women had higher oral bioavailability. *ABCB1* variants were additionally evaluated. |
| **3.1.2 Quinine metabolism in vitro** | | | | | | |
| Zhou et al. (2019) (69) | Recombinant CYP3A4 enzymes (in vitro) | Quinine | *CYP3A4*1A* and 30 allelic variants | In vitro enzyme-kinetic study | Quinine 3-hydroxylation / intrinsic clearance | Of 30 variants, 23 had decreased activity, two increased activity, two activity similar to *CYP3A4*1A* and three no detectable activity. Clinical relevance requires in vivo confirmation. |
| **3.1.3.1 Artemether-lumefantrine metabolism in non-pregnant patients** | | | | | | |
| Staehli Hodel et al. (2013) (71) | Cambodia (n=125) and Tanzania (n=150); analysis included 143 of the 150 patients recruited in Tanzania for lumefantrine | Artemisinin-based combination therapies, including artemether-lumefantrine | *CYP3A4*1B*, *CYP3A5*3* and other *CYP/NAT2* variants | Clinical pharmacogenetic/pharmacokinetic study | Pharmacokinetic parameters of lumefantrine and other ACT partner drugs | No significant association was identified between *CYP3A4*1B* or *CYP3A5*3* and lumefantrine pharmacokinetic parameters. |
| Kiaco et al. (2017) (72) | Angola; 103 patients with uncomplicated *P. falciparum* malaria | Artemether-lumefantrine | *CYP3A4*1B*, *CYP3A5*3* and *ABCB1 c.3435C>T* | Clinical genetic association study | Day-28 treatment outcome / recurrent infection | *CYP3A4*1B* and *CYP3A5*3* were not significantly associated with outcome. *ABCB1 c.3435CC* was significantly more frequent among recurrence-free patients (p < 0.01). |
| Marwa et al. (2014) (73) | Tanzania; 256 indigenous participants | No antimalarial administered; relevance to artemisinin-based combinations | *CYP3A5*3*, *CYP3A4*1B* and additional CYP variants | Cross-sectional population pharmacogenetic study | Allele frequencies | *CYP3A5*3* and *CYP3A4*1B* allele frequencies were 16% and 78%, respectively. Pharmacokinetics and treatment outcomes were not assessed. |
| Thomford et al. (2024) (74) | Ghana; 52 patients with uncomplicated malaria treated with generic artemether-lumefantrine | Generic artemether-lumefantrine | *CYP3A5*1/*1* and other *CYP3A5* genotypes | Clinical efficacy and pharmacogenetic study | Day-3/day-7 lumefantrine and desbutyl-lumefantrine concentrations; therapeutic efficacy | *CYP3A5*1/*1* was associated with lower lumefantrine and desbutyl-lumefantrine concentrations than several comparator genotypes, although reported concentrations remained above the stated therapeutic threshold. |
| **3.1.3.2 Artemether-lumefantrine metabolism in pregnancy** | | | | | | |
| Mutagonda et al. (2017) (75) | Tanzania; 92 malaria-positive pregnant women | Artemether-lumefantrine | *CYP3A4*1B* and *CYP3A5*1, *3, *6, *7* | Prospective clinical pharmacogenetic/pharmacokinetic study | Day-7 lumefantrine concentration and day-28 ACPR^1^ | *CYP3A5*3/*6/*7* genotypes were associated with higher day-7 lumefantrine concentrations than *CYP3A5*1/*1*. *CYP3A4*1B/*1B* was associated with a higher proportion of ACPR^1^ than *CYP3A4*1/*1*. |
| Mutagonda et al. (2019) (76) | Tanzania; 205 pregnant and 72 non-pregnant women | Artemether-lumefantrine | *CYP3A4*, *CYP3A5* and *ABCB1*; particularly *CYP3A5*1/*1* | Prospective observational pharmacogenetic study | Day-7 lumefantrine concentration and PCR-corrected day-28 ACPR^1^ | Pregnancy, low body weight and *CYP3A5*1/*1* independently predicted lower day-7 lumefantrine exposure (p < 0.01). Lower day-7 concentration was associated with treatment failure; ACPR^1^ was 93.0% in pregnant and 95.7% in non-pregnant women. |
| **3.1.3.3 Artemether-lumefantrine metabolism in HIV patients** | | | | | | |
| Maganda et al. (2016) (78) | Tanzania; 269 HIV-malaria co-infected patients in efavirenz, nevirapine and no-ART^2^ arms | Artemether-lumefantrine with efavirenz- or nevirapine-based ART^2^ | *CYP2B6*6/*18*, *CYP3A4*1B*, *CYP3A5*3/*6/*7* and *ABCB1* variants | Prospective clinical pharmacogenetic drug-interaction study | Day-7 lumefantrine concentration and recurrent parasitaemia | Efavirenz, high efavirenz concentrations and *CYP2B6*6/*6* were associated with lower lumefantrine exposure and more recurrent parasitaemia. Nevirapine had no significant effect; *CYP3A4/CYP3A5* variants were not the principal determinants. |
| Adegbola et al. (2020) (79) | Nigeria; 69 HIV-malaria co-infected women receiving efavirenz-based ART^2^ | Artemether-lumefantrine plus efavirenz | *CYP3A5*3* | Clinical pharmacokinetic/pharmacogenetic study | Lumefantrine Cmax and elimination half-life | *CYP3A5*3* was associated with higher maximum lumefantrine concentration. Day-7 lumefantrine concentration was not assessed. |
| Thomford et al. (2025) (83) | Ghana; HIV and malaria mono-infection and coinfection groups | Artemether-lumefantrine and dolutegravir | *CYP2B6*18*, *CYP3A4*1B* and *CYP3A5*3/*6/*7* | Clinical pharmacogenetic drug-drug interaction study | Day-7 lumefantrine and dolutegravir plasma concentrations | Coinfection was associated with lower day-7 plasma concentrations of lumefantrine (426.0 versus 741.5 ng/mL) and dolutegravir. Lower drug concentrations were observed in carriers of *CYP2B6*18*, *CYP3A4*1B,* and *CYP3A5*3, *6,* and **7* variant alleles. No clinically significant adverse reactions were reported. |
| **3.1.3.4 Artemether-lumefantrine metabolism in patients with tuberculosis** | | | | | | |
| *No direct pharmacogenetic study has evaluated CYP3A variants during concomitant artemether-lumefantrine and rifampicin-based tuberculosis treatment.* | | | | | | |
| **3.1.4 Artemether-lumefantrine metabolism in vitro** | | | | | | |
| Zhang et al. (2023) (86) | Recombinant CYP3A4 enzymes (in vitro) | Artemether and lumefantrine | *CYP3A4*1* and multiple allelic variants | In vitro enzyme-kinetic study | Allele-specific artemether and lumefantrine metabolism | Several variants decreased artemether metabolism. Lumefantrine metabolism varied bidirectionally; *CYP3A4*18, *23, *28 and *31-*34* showed opposite functional patterns for the two substrates. |
| **3.2.1 Primaquine** | | | | | | |
| Pybus et al. (2013) (55) | In vitro CYP2D6 systems and Plasmodium berghei mouse models | Primaquine | CYP2D6 inhibition, CYP2D knockout and humanised CYP2D6 | Experimental mechanistic study | Phenolic metabolite formation and causal prophylactic efficacy | Paroxetine inhibited CYP2D6-dependent metabolite formation. Primaquine had no efficacy in CYP2D-knockout mice at 20 or 40 mg/kg, while efficacy was partly restored in humanised CYP2D6 mice, demonstrating CYP2D6-dependent activation. |
| Bennett et al. (2013) (90) | USA; 33 malaria-naive adults in a *P. vivax* sporozoite challenge study; treatment failures analysed as clinical cases | Chloroquine 1500 mg base/48 h plus primaquine 30 mg/day for 14 days | *CYP2D6* reduced-function genotypes/phenotypes | Clinical treatment-failure report | Relapse after primaquine radical cure | Primaquine failure was reported in individuals with impaired CYP2D6 activity, providing early human evidence that reduced CYP2D6 function can compromise radical cure. |
| Baird et al. (2018) (91) | Indonesia; 57 patients (21 relapse cases, 36 controls) following directly observed primaquine radical cure for latent *P. vivax* infection | Primaquine | *CYP2D6* genotype-predicted and measured activity | Clinical pharmacogenetic efficacy study | Recurrence following primaquine treatment | Impaired *CYP2D6* genotype and phenotype were associated with reduced primaquine therapeutic efficacy and increased recurrence. |
| Silvino et al. (2020) (92) | Brazilian Amazon; 261 malaria-exposed individuals in a 10-year retrospective study | Primaquine | Reduced versus normal CYP2D6 activity phenotype | Retrospective pharmacogenetic cohort study | Number and risk of *P. vivax* recurrences | Reduced CYP2D6 activity was associated with a higher risk of multiple recurrences (risk ratio 1.75; 95% CI^4^ 1.2-2.6; p = 0.0035). Each additional year of malaria exposure reduced recurrence risk by about 3%. |
| de Pina-Costa et al. (2021) (93) | Brazil; three patients with recurrent *P. vivax* malaria | Chloroquine plus primaquine | CYP2D6 AS^3^ ≤ 1.0 (one PM^7^, one IM^5^ and one normal-slow metaboliser) | Case series | Relapse after standard treatment and response to increased primaquine dose | All three patients had impaired CYP2D6 activity. Increasing the total primaquine dose prevented further relapse in two; the third required additional weekly chloroquine. |
| Zeng et al. (2025) (94) | Northeastern Myanmar; 239 prospectively enrolled patients; nested case-control analysis (49 recurrent/49 non-recurrent) | Chloroquine plus 14-day primaquine (3.5 mg/kg total), directly observed | CYP2D6 activity score; AS^3^ ≤ 1.25 | Prospective efficacy cohort with nested case-control pharmacogenetic analysis | *P. vivax* recurrence during one-year follow-up | Patients with AS^3^ ≤ 1.25 had higher recurrence risk (OR^6^ 6.53; 95% CI^4^ 2.0-21; p < 0.001). More than 75% carried alleles predicting AS^3^ ≤ 1.25; one-year cumulative recurrence risk was 25.6%. |
| da Silva et al. (2025) (95) | Brazilian Amazon; 72 patients (18 recurrence cases, 54 non-recurrence controls) | Primaquine-based radical cure | *CYP2D6, MAOA* and *UGT2B7* variants | Clinical genetic association study | Risk and time to *P. vivax* recurrence | *CYP2D6*4* was more frequent among recurrence cases (p = 0.019), while CYP2D6 normal-metaboliser phenotypes were more frequent in non-recurrent cases (p = 0.039). *MAOA CC/CT* genotypes were associated with a shorter time to recurrence (p = 0.0437). No significant association was observed for *UGT2B7* |
| Sierra-Cifuentes et al. (2025) (96) | Turbo, Colombia; 78 *P. vivax* patients receiving chloroquine-primaquine | Chloroquine plus primaquine | CYP2D6 AS^3^ ≤ 1.25 versus normal/ultrarapid metabolism | Clinical pharmacogenetic/pharmacokinetic study | Day-2 primaquine concentration and relapse | Reduced-activity phenotypes had numerically higher day-2 primaquine concentrations, although the difference was not statistically significant; relapse risk was also not significantly higher than in normal/ultrarapid metabolisers. Approximately 18% had a reduced-activity phenotype. |
| **3.2.2 Tafenoquine** | | | | | | |
| St Jean et al. (2016) (63) | Multinational Phase 2b DETECTIVE trial population | Tafenoquine plus chloroquine | CYP2D6 reduced-metabolism phenotype | Post hoc pharmacogenetic analysis of a randomised trial | *P. vivax* relapse | Reduced CYP2D6 metabolism was not associated with relapse following tafenoquine; the evidence was described as suggestive because of limited numbers in reduced-metabolism groups. |
| Dowd et al. (2023) (61) | Australian Defence Force personnel; n=92 (31 relapse, 61 non-relapse) | Tafenoquine | *CYP2D6* profiles | Retrospective clinical pharmacogenetic efficacy analysis | Anti-relapse efficacy against *P. vivax* | *CYP2D6* profile was not associated with reduced tafenoquine anti-relapse efficacy. |
| Godinho et al. (2026) (102) | Manaus, Brazil; 118 P. vivax patients: primaquine n=67 (37 recurrence/30 controls), tafenoquine n=51 (16 recurrence/35 controls) | Tafenoquine or primaquine | *CYP2D6* variants | Comparative clinical pharmacogenetic study | *P. vivax* recurrence | *CYP2D6* variants had differential associations by drug: reduced CYP2D6 function was linked to primaquine recurrence, whereas tafenoquine recurrence was not associated with reduced CYP2D6 activity. |

1**)**ACPR= adequate clinical and parasitological response, 2)ART= antiretroviral therapy, 3)AS= activity score, 4)CI= confidence interval, 5)IM= intermediate metaboliser phenotype; 6)OR= odds ratio, 7)PM= poor metaboliser phenotype
